# *N*-Alkoxyphenylhydroxynaphthalenecarboxamides and Their Antimycobacterial Activity

**DOI:** 10.3390/molecules21081068

**Published:** 2016-08-16

**Authors:** Tomas Gonec, Sarka Pospisilova, Tereza Kauerova, Jiri Kos, Jana Dohanosova, Michal Oravec, Peter Kollar, Aidan Coffey, Tibor Liptaj, Alois Cizek, Josef Jampilek

**Affiliations:** 1 Department of Chemical Drugs, Faculty of Pharmacy, University of Veterinary and Pharmaceutical Sciences, Palackeho 1, Brno 61242, Czech Republic; jurd@email.cz; 2 Department of Infectious Diseases and Microbiology, Faculty of Veterinary Medicine, University of Veterinary and Pharmaceutical Sciences, Palackeho 1, Brno 61242, Czech Republic; sharka.pospisilova@gmail.com (S.P.); cizeka@vfu.cz (A.C.); 3 Department of Human Pharmacology and Toxicology, Faculty of Pharmacy, University of Veterinary and Pharmaceutical Sciences, Palackeho 1, Brno 61242, Czech Republic; tereza.kauerova@gmail.com (T.K.); kollarp@vfu.cz (P.K.); 4 Central Laboratories, Faculty of Chemical and Food Technology, Slovak University of Technology in Bratislava, Radlinskeho 9, Bratislava 81237, Slovakia; jana.dohanosova@stuba.sk (J.D.); tibor.liptaj@stuba.sk (T.L.); 5 Global Change Research Institute CAS, Belidla 986/4a, Brno 60300, Czech Republic; oravec.m@czechglobe.cz; 6 Department of Biological Sciences, Cork Institute of Technology, Bishopstown, Cork, Ireland; aidan.coffey@cit.ie; 7 Department of Pharmaceutical Chemistry, Faculty of Pharmacy, Comenius University, Odbojarov 10, Bratislava 83232, Slovakia

**Keywords:** hydroxynaphthalenecarboxamides, in vitro antimycobacterial activity, MTT assay, lipophilicity, structure-activity relationships

## Abstract

A series of nineteen *N*-(alkoxyphenyl)-2-hydroxynaphthalene-1-carboxamides and a series of their nineteen positional isomers *N*-(alkoxyphenyl)-1-hydroxynaphthalene-2-carboxamides were prepared and characterized. Primary in vitro screening of all the synthesized compounds was performed against *Mycobacterium tuberculosis* H37Ra, *M. kansasii* and *M. smegmatis*. Screening of the cytotoxicity of the compounds was performed using human monocytic leukemia THP-1 cells. Some of the tested compounds showed antimycobacterial activity comparable with or higher than that of rifampicin. For example, 2-hydroxy-*N*-(4-propoxyphenyl)-naphthalene-1-carboxamide showed the highest activity (MIC = 12 µM) against *M. tuberculosis* with insignificant cytotoxicity. *N*-[3-(But-2-yloxy)phenyl]- and *N*-[4-(but-2-yloxy)phenyl]-2-hydroxy-naphthalene-1-carboxamide demonstrated high activity against all tested mycobacterial strains and insignificant cytotoxicity. *N*-(Alkoxyphenyl)-1-hydroxynaphthalene-2-carboxamides demonstrated rather high effect against *M. smegmatis* and *M. kansasii* and strong antiproliferative effect against the human THP-1 cell line. Lipophilicity was found as the main physicochemical parameter influencing the activity. A significant decrease of mycobacterial cell metabolism (viability of *M. tuberculosis* H37Ra) was observed using MTT (3-(4,5-dimethylthiazol-2-yl)-2,5-diphenyl-tetrazolium bromide) assay. Structure-activity relationships are discussed.

## 1. Introduction

In spite of the approval of some new antituberculosis drugs, such as bedaquillin or delamanid [1], tuberculosis (TB) now ranks alongside human immunodeficiency virus as a leading cause of deaths worldwide [2]. Despite the decrease in TB incidence since the 1950s due to the introduction of new antitubercular agents to clinical practice, morbidity and mortality have risen again since the 1980s; TB has again become a major bacterial cause of worldwide mortality, and thus it remains a serious global problem. Based on the new World Health Organization tuberculosis report, TB killed 1.5 million people in 2014 worldwide; 9.6 million people are estimated to have fallen ill with TB in 2014. As estimated, 480,000 cases of multidrug-resistant TB (MDR-TB) occurred in 2014 [2]. The rate of successful treatments has also decreased due to the emergence of drug resistant, cross-, multidrug-, extensively- and totally-resistant strains. The increase in the number of new infections is also associated with immunocompromised populations. In addition, more frequent occurrences of lethal complications associated with immunocompromised populations include systemic infections caused by common, initially non-pathogenic mycobacterial strains (e.g., *M. kansasii*, *M. avium*, *M. smegmatis*, etc.), which cause difficult-to-treat or incurable diseases due to suppressed immunity. These non-tuberculous mycobacteria (NTM) are now recognized as significant human pathogens and cause diseases (such as pulmonary disease, lymphadenitis, skin and soft tissue disease, gastrointestinal and skeletal infections) that result in significant morbidity. The emergence of MDR-TB and NTM makes the discovery of new molecular scaffolds a priority to achieve effective control of both TB and NTM [3].

Hydroxynaphthalene-2-carboxanilides can be considered as cyclic analogues of salicylanilides that have expressed promising results as potential antimicrobial and antimycobacterial agents [4,5,6] (and refs. therein)]. Their antimicrobial effects are connected with the ability of vicinal hydroxyarylamides to inhibit various enzymatic systems in bacteria [[4,5,6] (and refs. therein)]. In addition, the presence of an amide group with a hydrophobic residue in its close vicinity is able, due to its electron properties, to interact and bind with a number of enzymes/receptors and affect the biological response by means of these target sites. The properties of the amide moiety can be easily modified by various substitutions [7]. Thus the presence of an amide-like moiety is characteristic for various antibacterial, antimycobacterial or antiparasitic agents [4,5,6,7,8,9,10,11,12,13,14,15].

*N*-Aryl-2-hydroxynaphthalene-1-carboxamides and *N*-aryl-1-hydroxynaphthalene-2-carbox-amides have shown activity against mycobacterial species [11,16]; therefore various alkoxy derivatives have been designed from 2-hydroxynaphthalene-1-carboxylic acid and 1-hydroxynaphthalene-2-carboxylic acid. The present work is focused on synthesis and investigation of the biological activity of ring-substituted carboxamides of the above-mentioned acids as promising antimycobacterial agents.

## 2. Results and Discussion

### 2.1. Chemistry and Physicochemical Properties

**Scheme 1:**
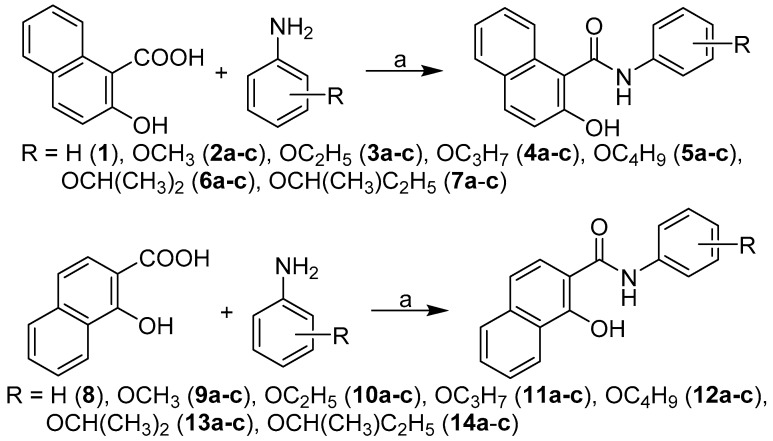
Synthesis of *N*-(alkoxyphenyl)-2-hydroxynaphthalene-1-carboxamides **1**–**7c** and *N*-(alkoxyphenyl)-1-hydroxynaphthalene-2-carboxamides **8**–**14c**. *Reagents and conditions*: (**a**) PCl_3_, chlorobenzene, microwave irradiation (MW).

The condensation of 2-hyroxynaphthalene-1-carboxylic acid or 1-hydroxynaphthalene-2-carboxylic acid with appropriate alkoxy-substituted anilines using phosphorus trichloride in dry chlorobenzene under microwave conditions yielded a series of nineteen *N*-substituted-2-hydroxy-naphthalene-1-carboxamides **1**–**7c** (series I) and nineteen *N*-substituted-1-hydroxynaphthalene-2-carboxamides **8**–**14c** (series II), see Scheme 1. Alkoxy-substituted anilines (except commercially available *o*-, *m*- and *p*-anisidine) were prepared from corresponding aminophenol and alkyl bromide according to De Marco et al. [17] and were reported recently [18]. Compounds **1**–**2c** [16] and **8**–**9c** [11] have been already published by our team, nevertheless they are also mentioned here to complete the overview of biological activities and structure-activity relationships.

Within structure-activity relationships, various parameters describing physicochemical properties are investigated. Lipophilicity is a property that has a major effect on solubility, absorption, distribution and biotransformation as well as pharmacological activity, because drugs cross biological membranes through passive transport, which strongly depends on their lipophilicity. Lipophilicity was observed as the most important descriptor affecting the antimycobacterial activity for both series, see below. In the current investigation, the lipophilicity values expressed as log *P* were calculated using ACD/Percepta ver. 2012. Log *P* values of both series as well as the used standards isoniazid (INH) and rifampicin (RIF) can be compared in Table 1. In general, *N*-(alkoxyphenyl)-1-hydroxynaphthalene-2-carboxamides **8**–**14c** (series II) are characterized by slightly higher calculated lipophilicity than *N*-(alkoxyphenyl)-2-hydroxynaphthalene-1-carbox-amides **1**–**7c** (series I) with the exception of compound **3c** (R = 4-OC_2_H_5_) that has higher log *P* value than compound **10c**. Within individual series, the lipophilicity increases as follows: OCH_3_ < OC_2_H_5_ < OCH(CH_3_)_2_ < OC_3_H_7_ < OCH(CH_3_)CH_2_CH_3_ < OC_4_H_9_. The *ortho*-substituted derivatives showed the highest calculated log *P* values, while *para*-substituted derivatives demonstrated the lowest log *P* values, except **11c** (R = 4-OC_3_H_7_) and **12c** (R = 4-OC_4_H_9_) that have the same calculated lipophilicity values as the *ortho*-substituted derivatives. In addition to lipophilicity, molar volume (MV) of substituents and electronic σ parameters of substituents are largely employed in structure-activity relationship analysis. The corresponding MV and σ values of phenyl ring substituents **1**–**14c** are shown in Table 1 and were also predicted by ACD/Percepta.

### 2.2. In Vitro Antimycobacterial Evaluation

The evaluation of the in vitro antimycobacterial activity of the compounds was performed against *Mycobacterium tuberculosis* H37Ra ATCC 25177 (MT), *M. kansasii* DSM 44162 (MK) and *M. smegmatis* ATCC 700084 (MS), see Table 1. To lower risk and make manipulation in the laboratory easier, surrogate model pathogens for *M. tuberculosis* can be used in laboratory studies. Avirulent *M. tuberculosis* strain H37Ra is very closely related to and has similar pathology as human-infecting *M. tuberculosis* strains, making it a good model for study especially because of the lower risk for laboratory workers [19]. *M. kansasii*, the most virulent of the NTM, causes nontuberculous mycobacterial lung infections, which are now very common, and can be indistinguishable from tuberculosis [20]. *M. smegmatis* is an ideal representative of a fast-growing nonpathogenic microorganism, particularly useful in studying basic cellular processes of special relevance to pathogenic mycobacteria [21]. Therefore additionally *M. kansasii* and *M. smegmatis* were chosen as model species for screening of prospective antimycobacterial drugs to control mycobacterial diseases. The potency of the compounds was expressed as the minimum inhibitory concentration (MIC) that is defined for mycobacteria as 90% or greater (IC_90_) reduction of growth in comparison with the control.

Compounds series I and II can be considered as positional isomers that differ from each other by the electron density of carboxamide and phenolic moieties as well as by the steric properties of the entire scaffolds. Nevertheless, in general, compounds **1**–**7c** demonstrated higher potency against *M. tuberculosis*, while compounds **8**–**14c** showed higher effect against the two NTM strains. Unfortunately, compounds of series II showed higher antiproliferative effect on human cells, see Table 1 and Section 2.3.

**Figure 1:**
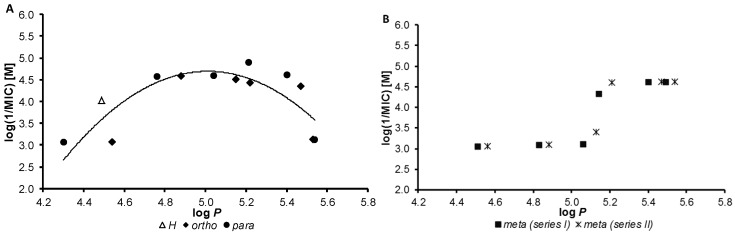
Relationships between in vitro antitubercular activity against *M. tuberculosis* log(1/MIC (mol/L)) and lipophilicity expressed as log *P* of unsubstituted, *ortho*- and *para*-substituted derivatives of series I (**A**) and *meta*-substituted derivatives of series I and II (**B**).

The dependences of the antitubercular activity of the compounds against *M. tuberculosis* expressed as log (1/MIC (M)) on lipophilicity expressed as log *P* are illustrated in Figure 1A,B. Similar trends can be observed also for dependences of activities on molar volume (MV) of R substituents; therefore they are not illustrated. Based on Figure 1A, it can be stated that compounds of series I in the range of log *P* values from ca. 4.8 to 5.5 showed potency against *M. tuberculosis*. The quasi-parabolic dependences of activity on log *P* (with log *P* optimum ca. 5) for the *ortho*- and *para*-substituted derivatives can be observed. The unexpected significant activity slump of both butoxy derivatives **5a** and **5c** can be caused by a steric hindrance or limited solubility. On the other hand, for *meta*-substituted derivatives of series I, the activity increased up to log *P* ca. 5.1 (**4b**, R = 3-OC_3_H_7_), see Figure 1B, and then only insignificantly increased up to 5.49 (compound **5b**) with increasing log *P*. It can be only speculated whether the activity would remain constant or it would decrease with a further prolongation of the alkoxy chain. Only 3-propoxy (**11b**), 3-butoxy (**12b**) and 3-*sec*-butoxy (**14b**) substituted compounds of series II showed activity comparable with that of series I. In general, the *meta*-substituted derivatives of series II demonstrated the same course of dependence (activity increase from log *P* ca. 5.2) as the *meta*-substituted derivatives of series I, see Figure 1B. The *ortho*- and *para*-substituted derivatives of series II expressed no effect; therefore they are not illustrated. It is important to note that the dependence of the antitubercular activity of the *ortho*- and *para*-substituted derivatives on electronic σ parameters was not observed; the antitubercular activity of the *meta*-substituted derivatives of series I and II is influenced by electron-donor properties of the substituents: σ = 0.14 (compounds **4b**, **5b**, **7b**) vs. σ ≈ 0.10 of the rest of ineffective C_(3)_′ substituted compounds.

Additionally, a standard MTT assay was performed on the selected most effective compounds against *M. tuberculosis* H37Ra, the MICs of which were previously determined through alamarBlue assays, see Table 1. The MTT assay is a well-characterized method of assessing cell growth through measurement of respiration. For the purpose of this assay, a positive result was recorded when the MTT measured viability of *M. tuberculosis* H37Ra was less than 70% after exposure to the MIC of each test compound. As such, a low level of cell viability may suggest inhibition of cell growth through respiratory inhibition [22]. All the selected compounds, i.e., **3a** (R = 2-OC_2_H_5_, 30.6%), **3c** (R = 4-OC_2_H_5_, 9.96%), **4c** (R = 4-OC_3_H_7_, 5.90%), **5b** (R = 3-OC_4_H_9_, 7.38%), **6c** (R = 4-OCH(CH_3_)_2_, 8.96%), **7b** (R = 3-OCH(CH_3_)CH_2_CH_3_, 7.38%), **7c** (R = 4-OCH(CH_3_)CH_2_CH_3_, 8.86%), **11b** (R = 3-OC_3_H_7_, 15.13%), **12b** (R = 3-OC_4_H_9_, 9.96%) and **14b** (R = 3-OCH(CH_3_)CH_2_CH_3_, 9.96%) showed less than 70% viability of *M. tuberculosis* H37Ra at the tested concentration equal to MICs.

**Figure 2:**
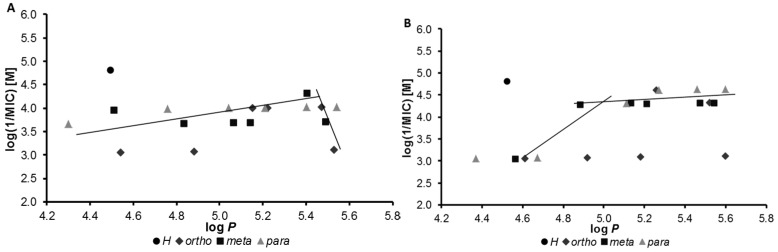
Relationships between in vitro antimycobacterial activity against *M. kansasii* log (1/MIC (mol/L)) and lipophilicity expressed as log *P* of compounds **1**–**7c** (**A**) and compounds **8**–**14c** (**B**).

Unsubstituted compounds **1** and **8** showed the highest effect against *M. kansasii*, nevertheless, it can be stated that in general, the compounds of series II expressed higher potency against *M. kansasii* than compounds of series I. The bilinear dependence can be found for compounds of series I, see Figure 2A, where relationships between the activity against *M. kansasii* and the lipophilicity of compounds are illustrated. The activity increases with increasing lipophilicity to the optimum of log *P* ca. 5.4, and then a significant activity decrease for butoxy derivatives can be found, similarly as mentioned above. On the other hand, the effect of the *meta*- and *para*-substituted derivatives of series II increases rapidly up to log *P* ca. 5, and then the activity is approximately linear, see Figure 2B. Similar insignificant influences of *meta*- and *para*-substituents were observed also by Kos et al. [14,15]. Among the *ortho*-substituted derivatives of series II, only **11a** (R = 2-OC_3_H_7_) and **14a** (R = 2-OCH(CH_3_)CH_2_CH_3_) demonstrated an activity at log *P* ca. 5.3. The dependences of activities on the molar volume of R substituents are not illustrated due to their similarity with dependences on lipophilicity.

The compounds of both series showed only moderate activity against *M. smegmatis*, except derivatives substituted by a *sec*-butoxy chain in C_(3)_′ and C_(4)_′ positions (compounds **7b**, **7c**, **14b** and **14c**). Because especially *meta*-substituted compounds **7b** and **14b** showed good activity against *M. tuberculosis* and *M. kansasii*, it can be assumed that the physicochemical and geometrical properties of the branched *sec*-butoxy chain are advantageous from the aspect of mycobacteria growth inhibition.

As mentioned above, the discussed compounds are cyclic analogues of salicylanilides. The phenolic moiety spatially close to the amide group forms a hypothetic six-membered ring by *H*-bonds with amide nitrogen, and thus a stable hydrophobic coplanar anionic form with a delocalized negative charge is generated, which seems to be decisive in the proton motive force. This enables the molecule to penetrate biological membranes not only as protonated neutral compound but also in the charged state. Thus salicylanilides are able to induce the uncoupling of oxidative phosphorylation and photophosphorylation in mitochondria, chloroplasts and other energy-transducing membranes [4,23,24,25,26]; therefore also the discussed structures could be considered as compounds that affect the proton motive force across membranes (uncouplers) [4,25,26,27,28]. The *ortho*-substituted derivatives of series I showed potency especially against *M. tuberculosis*. The broader antimycobacterial activity of the *meta*- and *para*-substituted derivatives in comparison with the *ortho*-substituted compounds can be related to the steric effect of the spatially close *ortho*-substituents [29]. A moiety in the *ortho* position results in a change of the molecule planarity [30], and different electron densities at the carbonyl and consequently at the phenolic moiety, which can break the formation of coplanar anionic form with a delocalized negative charge (i.e., uncoupler effect) and influence the potential of binding of the carboxamide and the phenolic groups to possible targets (sensitive enzymatic systems affecting proliferative cell functions as mentioned in Introduction) in a mycobacterial cell. In the case of the *meta*- and *para*-derivatives, the described secondary steric effect did not manifest [18,29,30]. The *para*-substituted as well as the *meta*-substituted derivatives should have practically a linear/planar structure as was, for example, described for a similar type of molecule, where the structural analysis displayed a planar structure [31,32,33,34]. Thus, it seems that the mode of action of this type of compounds demonstrates multitarget activity that should not be very different from that of other planar antimycobacterial agents [4,6,24,30,35,36,37]. The lipophilicity of *meta*- and *para*-substituted derivatives was found as the factor conducive to the activity of such structures. More lipophilic structures displayed relatively higher efficiency, and simultaneously planar structures easier permeate through various types of membranes.

### 2.3. In Vitro Antiproliferative Assay

The preliminary in vitro screening of the antiproliferative activity of the most effective antimycobacterial compounds as well as both standards was performed using Water Soluble Tetrazolium Salts-1 (WST-1) assay kit [38] and the human monocytic leukemia THP-1 cell line by means of the method described recently [14]. The antiproliferative activity was evaluated as the IC_50_ value (compound concentration causing 50% inhibition of cell population proliferation), see Table 1. A compound is considered as cytotoxic when it demonstrates a toxic effect on cells at the concentration up to 10 μM [39], and the highest tested concentration that was used for the toxicity assay was 3-fold this value.

As mentioned above, the compounds of series I showed lower antiproliferative effect than the compounds of series II, see Table 1. The antiproliferative-efficient compounds inhibit mitochondrial dehydrogenases (the principle of WST-1 assay kit), the activity of which directly correlates with the number of metabolically active cells in the culture. All the compounds from series I effective against *M. tuberculosis* showed IC_50_ > 30 µM, i.e., the treatment with this concentration did not lead to significant antiproliferative effect on THP-1 cells, and compounds **3a**, **4c**, **5b**, **7b** and **7c** inhibited selectively vital processes in *M. tuberculosis*. Based on these observations, it can be concluded that the discussed amides can be considered as non-toxic agents for subsequent design of novel potential antitubercular agents. Unfortunately, the potency of these compounds against NTM strains is medium or moderate, except for compound **7b**. The antimycobacterial potency of compounds from series II is connected with their rather nonselective prokaryote and eukaryote antiproliferative effect; IC_50_ ranged from 1.2 to 9.4 µM (for comparison, e.g., IC_50_ of camptothecin, assessed in this line formerly, was 0.16 ± 0.07 µM), except for compounds **8** (R = H), **11c** (R = 4-OC_3_H_7_) and **12c** (R = 4-OC_4_H_9_) with IC_50_ > 30 µM, see Table 1. These can be considered as compounds with noteworthy effect against *M. kansasii*.

## 3. Experimental Section

### 3.1. General Information

All reagents were purchased from Sigma-Aldrich (St. Louis, MO, USA) or Merck (Darmstadt, Germany). Reactions were carried out in a StartSYNTH microwave labstation (Milestone, Sorisole, Italy). Melting points were determined on a Kofler hot plate apparatus HMK (Franz Kustner Nacht GK, Dresden, Germany) and left uncorrected. Column chromatography was performed on a 200 mL column (Sigma-Aldrich) using Silica gel 60 (0.040–0.063 mm, Merck). Infrared (IR) spectra were recorded on a Smart MIRacle™ ATR ZnSe for Nicolet™ Impact 400 FT-IR spectrometer (Thermo Fisher Scientific, West Palm Beach, FL, USA). All ^1^H- and ^13^C-NMR spectra were recorded on an Agilent VNMRS 600 MHz system (600 MHz for ^1^H and 150 MHz for ^13^C, Agilent Technologies, Santa Clara, CA, USA) equipped with a triple resonance HCN probe at 25 °C in DMSO-*d*_6_. Chemical shifts are reported in ppm (δ) using the signal of the solvent (DMSO; δ(^1^H) = 2.5 ppm, δ(^13^C) = 49.5 ppm) for referencing. High-resolution mass spectra were measured using a high-performance liquid chromatograph Dionex UltiMate^®^ 3000 (Thermo Fisher Scientific) coupled with an LTQ Orbitrap XL™ Hybrid Ion Trap-Orbitrap Fourier Transform Mass Spectrometer (Thermo Fisher Scientific) with injection into HESI II in the positive mode.

### 3.2. Synthesis

#### 3.2.1. General Procedure for Synthesis of *N*-(Alkoxyphenyl)-2-hydroxynaphthalene-1-carboxamides **1**–**7c** and *N*-(Alkoxyphenyl)-1-hydroxynaphthalene-2-carboxamides **8**–**14c**

2-Hydroxynaphthalene-1-carboxylic acid or 1-hydroxynaphthalene-2-carboxylic acid (5.30 mmol) and appropriate alkoxyaniline (5.30 mmol) were suspended in 30 mL of dry chlorobenzene. Phosphorous trichloride (2.65 mmol) was added dropwise, and reacting mixture was heated in the microwave reactor for 15 min at 130 °C using infrared flask-surface control of temperature. Solvent was evaporated in vacuum; residue solid was washed with 2M HCl and crystallized from aqueous ethanol. If necessary, column chromatography was used for further purification (mobile phase DCM:MeOH 19:1).

*2-Hydroxy-N-phenylnaphthalene-1-carboxamide* (**1**, CAS 16670-63-6), *2-hydroxy-N-(2-methoxyphenyl)naphtha-lene-1-carboxamide* (**2a**, CAS 337955-96-1), *2-hydroxy-N-(3-methoxyphenyl)naphthalene-1-carboxamide* (**2b**, CAS 1463483-65-9) and *2-hydroxy-N-(4-methoxyphenyl)naphthalene-1-carboxamide* (**2c**, CAS 856062-47-0) were synthesized and characterized recently [16].

*N-(2-Ethoxyphenyl)-2-hydroxynaphthalene-1-carboxamide* (**3a**, CAS 858031-71-7). Yield 62%; Mp 116 °C (155–157 °C) [40]; IR (cm^−1^): 3318 (ν NH), 1623 (ν C=O), 1539 (δ NH), 1391 (δ COH), 1284 (ν CN), 1118 (ν C_Ar_CO); ^1^H-NMR (DMSO-*d*_6_), δ: 10.48 (br. s, 1H, OH), 9.43 (s, 1H, NH), 8.19 (d, *J* = 9.2 Hz, 1H, ArH_Phe_), 8.15 (d, *J* = 9.2 Hz, 1H, ArH_Naph_), 7.88 (d, *J* = 8.4 Hz, 1H, ArH_Naph_), 7.84 (d, *J* = 7.0 Hz, 1H, ArH_Naph_), 7.48 (td, *J* = 8.4 Hz, *J* = 1.1 Hz, 1H, ArH_Naph_), 7.34 (td, *J* = 8.1 Hz, *J* = 1.1 Hz, 1H, ArH_Naph_), 7.24 (d, *J* = 9.2 Hz, 1H, ArH_Naph_), 7.13–6.96 (m, 3H, ArH_Phe_), 4.10 (q, *J* = 7.0 Hz, 2H, CH_2_), 1.36 (t, *J* = 7.0 Hz, 3H, CH_3_); ^13^C-NMR (DMSO-*d*_6_), δ: 165.06, 152.42, 148.91, 131.81, 131.05, 127.94, 127.73, 127.73, 126.85, 124.50, 124.12, 123.03, 121.74, 120.33, 118.25, 116.60, 112.21, 63.99, 14.57; HR-MS: for C_19_H_17_NO_3_ [M + H]^+^ calculated 308.12812 *m*/*z*, found 308.12827 *m*/*z*.

*N-(3-Ethoxyphenyl)-2-hydroxynaphthalene-1-carboxamide* (**3b**). Yield 64%; Mp 133 °C; IR (cm^−1^): 3323 (ν NH), 1623 (ν C=O), 1536 (δ NH), 1387 (δ COH), 1284 (ν CN), 1158 (ν C_Ar_CO); ^1^H-NMR (DMSO-*d*_6_), δ: 10.34 (s, 1H, NH), 10.05 (br. s, 1H, OH), 7.85 (d, *J* = 8.8 Hz, 2H, ArH_Naph_), 7.68 (d, *J* = 8.1 Hz, 1H, ArH_Naph_), 7.53 (t, *J* = 2.0 Hz, 1H, ArH_Phe_), 7.46 (td, *J* = 7.6 Hz, *J* = 1.3 Hz, 1H, ArH_Naph_), 7.36–7.19 (m, 4H, ArH_Naph_, ArH_Phe_), 6.66 (ddd, *J* = 7.9 Hz, *J* = 2.2 Hz, *J* = 0.7 Hz, 1H, ArH_Phe_), 4.02 (q, *J* = 7.0 Hz, 2H, CH_2_), 1.34 (t, *J* = 7.0 Hz, 3H, CH_3_); ^13^C-NMR (DMSO-*d*_6_), δ: 165.68, 158.73, 151.55, 140.74, 131.36, 130.04, 129.34, 127.87, 127.35, 126.85, 123.35, 122.91, 118.58, 118.31, 111.53, 109.19, 105.66, 62.87, 14.60; HR-MS: for C_19_H_17_NO_3_ [M + H]^+^ calculated 308.12812 *m*/*z*, found 308.12869 *m*/*z*.

*2-Hydroxy-N-(2-propoxyphenyl)naphthalene-1-carboxamide* (**4a**). Yield 80%; Mp 125 °C; IR (cm^−1^): 3307 (ν NH), 1622 (ν C=O), 1537 (δ NH), 1390 (δ COH), 1286 (ν CN), 1117 (ν C_Ar_CO); ^1^H-NMR (DMSO-*d*_6_), δ: 10.49 (s, 1H, OH), 9.40 (s, 1H, NH), 8.20 (d, *J* = 9.2 Hz, 1H, ArH_Phe_), 8.15 (d, *J* = 9.2 Hz, 1H, ArH_Naph_), 7.88 (d, *J* = 8.8 Hz, 1H, ArH_Naph_), 7.84 (d, *J* = 7.0 Hz, 1H, ArH_Naph_), 7.48 (td, *J* = 7.3 Hz, *J* = 1.3 Hz, 1H, ArH_Naph_), 7.34 (td, *J* = 7.9 Hz, *J* = 1.1Hz, 1H, ArH_Naph_), 7.28 (d, *J* = 9.2 Hz, 1H, ArH_Naph_), 7.16–6.95 (m, 3H, ArH_Phe_), 4.00 (t, *J* = 6.4 Hz, 2H, CH_2_), 1.76 (sx, *J* = 6.8 Hz, 2H, CH_2_), 0.97 (t, *J* = 7.3 Hz, 3H, CH_3_); ^13^C-NMR (DMSO-*d*_6_), δ: 165.04, 152.45, 149.03, 131.81, 131.10, 127.96, 127.75, 127.63, 126.85, 124.52, 124.11, 123.04, 121.71, 120.27, 118.23, 116.53, 112.09, 69.76, 21.98, 10.35; HR-MS: for C_20_H_19_NO_3_ [M + H]^+^ calculated 322.14377 *m*/*z*, found 322.14438 *m*/*z*.

*2-Hydroxy-N-(3-propoxyphenyl)naphthalene-1-carboxamide* (**4b**). Yield 87%; Mp 125 °C; IR (cm^−1^): 3352 (ν NH), 1629 (ν C=O), 1538 (δ NH), 1321 (δ COH), 1276 (ν CN), 1182 (ν C_Ar_CO); ^1^H-NMR (DMSO-*d*_6_), δ: 10.34 (s, 1H, NH), 10.10 (br. s, 1H, OH), 7.85 (d, *J* = 8.8 Hz, 2H, ArH_Naph_), 7.68 (d, *J* = 8.4 Hz, 1H, ArH_Naph_), 7.55 (t, *J* = 1.3 Hz, 1H, ArH_Phe_), 7.46 (td, *J* = 7.1 Hz, *J* = 1.1 Hz, 1H, ArH_Naph_), 7.38–7.18 (m, 4H, ArH_Naph_, ArH_Phe_), 6.67 (dd, *J* = 8.1 Hz, *J* = 2.2 Hz, 1H, ArH_Phe_), 3.92 (t, *J* = 6.6 Hz, 2H, CH_2_), 1.74 (sx, *J* = 7.0 Hz, 2H, CH_2_), 0.99 (t, *J* = 7.3 Hz, 3H, CH_3_); ^13^C-NMR (DMSO-*d*_6_), δ: 165.69, 158.91, 151.55, 140.74, 131.36, 130.04, 129.33, 127.87, 127.35, 126.87, 123.35, 122.91, 118.58, 118.31, 111.50, 109.22, 105.70, 68.83, 21.98, 10.32; HR-MS: for C_20_H_19_NO_3_ [M + H]^+^ calculated 322.14377 *m*/*z*, found 322.14420 *m*/*z*.

*2-Hydroxy-N-(4-propoxyphenyl)naphthalene-1-carboxamide* (**4c**). Yield 71%; Mp 153 °C; IR (cm^−1^): 3328 (ν NH), 1627 (ν C=O), 1531 (δ NH), 1435 (δ COH), 1236 (ν CN), 1169 (ν C_Ar_CO); ^1^H-NMR (DMSO-*d*_6_), δ: 10.23 (s, 1H, NH), 10.00 (br. s, 1H, OH), 7.84 (d, *J* = 8.4 Hz, 2H, ArH_Naph_), 7.71 (d, *J* = 8.8 Hz, 2H, ArH_Phe_), 7.68 (d, *J* = 7.0 Hz, 1H, ArH_Naph_), 7.45 (td, *J* = 7.0 Hz, *J* = 1.1 Hz, 1H, ArH_Naph_), 7.32 (td, *J* = 8.0 Hz, *J* = 2.0 Hz, 1H, ArH_Naph_), 7.24 (d, *J* = 9.2 Hz, 1H, ArH_Naph_), 6.92 (d, *J* = 8.8 Hz, 2H, ArH_Phe_), 3.91 (t, *J* = 6.6 Hz, 2H, CH_2_), 1.73 (sx, *J* = 7.0 Hz, 2H, CH_2_), 0.99 (t, *J* = 7.3 Hz, 3H, CH_3_); ^13^C-NMR (DMSO-*d*_6_), δ: 165.07; 154.62, 151.51, 132.75, 131.45, 129.89, 127.84, 127.37, 126.76, 123.45, 122.85, 120.71, 118.69, 118.32, 114.38, 69.09, 22.02, 10.32; HR-MS: for C_20_H_19_NO_3_ [M + H]^+^ calculated 322.14377 *m*/*z*, found 322.14429 *m*/*z*.

*N-(2-Butoxyphenyl)-2-hydroxynaphthalene-1-carboxamide* (**5a**). Yield 70%; Mp 108 °C; IR (cm^−1^): 3301 (ν NH), 1621 (ν C=O), 1536 (δ NH), 1392 (δ COH), 1287 (ν CN), 1115 (ν C_Ar_CO); ^1^H-NMR (DMSO-*d*_6_), δ: 10.45 (s, 1H, OH), 9.37 (s, 1H, NH), 8.17 (d, *J* = 8.4 Hz, 1H, ArH_Phe_), 8.12 (d, *J* = 9.5 Hz, 1H, ArH_Naph_), 7.88 (d, *J* = 8.8 Hz, 1H, ArH_Naph_), 7.84 (d, *J* = 7.0 Hz, 1H, ArH_Naph_), 7.47 (td, *J* = 7.0 Hz, *J* = 1.1 Hz, 1H, ArH_Naph_), 7.34 (td, *J* = 7.9 Hz, *J* = 2.0 Hz, 1H, ArH_Naph_), 7.27 (d, *J* = 9.2 Hz, 1H, ArH_Naph_), 7.13–6.95 (m, 3H, ArH_Phe_), 4.04 (t, *J* = 6.4 Hz, 2H, CH_2_), 1.73 (qi, *J* = 6.9 Hz, 2H, CH_2_), 1.42 (sx, *J* = 7.4 Hz, 2H, CH_2_), 0.88 (t, *J* = 7.3 Hz, 3H, CH_3_); ^13^C-NMR (DMSO-*d*_6_), δ: 165.04, 152.42, 149.08, 131.75, 131.05, 127.94, 127.72, 127.61, 126.82, 124.55, 124.06, 123.03, 121.77, 120.27, 118.22, 116.62, 112.10, 67.95, 30.64, 18.58, 13.57; HR-MS: for C_21_H_21_NO_3_ [M + H]^+^ calculated 336.15942 *m*/*z*, found 336.15982 *m*/*z*.

*N-(3-Butoxyphenyl)-2-hydroxynaphthalene-1-carboxamide* (**5b**). Yield 46%; Mp 118 °C; IR (cm^−1^): 3350 (ν NH), 1628 (ν C=O), 1537 (δ NH), 1321 (δ COH), 1276 (ν CN), 1182 (ν C_Ar_CO); ^1^H-NMR (DMSO-*d*_6_), δ: 10.33 (s, 1H, NH), 10.10 (br. s, 1H, OH), 7.85 (d, *J* = 8.8 Hz, 2H, ArH_Naph_), 7.67 (d, *J* = 8.1 Hz, 1H, ArH_Naph_), 7.54 (t, *J* = 1.8 Hz, 1H, ArH_Phe_), 7.46 (td, *J* = 7.2 Hz, *J* = 1.1 Hz, 1H, ArH_Naph_), 7.36–7.18 (m, 4H, ArH_Naph_, ArH_Phe_), 6.69–6,65 (m, 1H, ArH_Phe_), 3.96 (t, *J* = 6.4 Hz, 2H, CH_2_), 1.72 (qi, *J* = 7.3 Hz, 2H, CH_2_), 1.45 (sx, *J* = 7.5 Hz, 2H, CH_2_), 0.94 (t, *J* = 7.3 Hz, 3H, CH_3_); ^13^C-NMR (DMSO-*d*_6_), δ: 165.68, 158.91, 151.55, 140.72, 131.36, 130.02, 129.33, 127.87, 127.34, 126.85, 123.35, 122.89, 118.57, 118.31, 111.51, 109.21, 105.67, 67.01, 30.68, 18.67, 13.60; HR-MS: for C_21_H_21_NO_3_ [M + H]^+^ calculated 336.15942 *m*/*z*, found 336.15963 *m*/*z*.

*N-(4-Butoxyphenyl)-2-hydroxynaphthalene-1-carboxamide* (**5c**). Yield 43%; Mp 158 °C; IR (cm^−1^): 3325 (ν NH), 1619 (ν C=O), 1532 (δ NH), 1435 (δ COH), 1238 (ν CN), 1170 (ν C_Ar_CO); ^1^H-NMR (DMSO-*d*_6_), δ: 10.22 (s, 1H, NH), 10.07 (br. s, 1H, OH), 7.84 (d, *J* = 8.8 Hz, 2H, ArH_Naph_), 7.71 (d, *J* = 9.2 Hz, 2H, ArH_Phe_), 7.68 (d, *J* = 7.3 Hz, 1H, ArH_Naph_), 7.46 (td, *J* = 7.3 Hz, *J* = 1.1 Hz, 1H, ArH_Naph_), 7.32 (td, *J* = 8.1 Hz, *J* = 1.1 Hz, 1H, ArH_Naph_), 7.24 (d, *J* = 9.2 Hz, 1H, ArH_Naph_), 6.92 (d, *J* = 9.2 Hz, 2H, ArH_Phe_), 3.95 (t, *J* = 6.4 Hz, 2H, CH_2_), 1.70 (qi, *J* = 6.2 Hz, 2H, CH_2_), 1.44 (sx, *J* = 6.6 Hz, 2H, CH_2_), 0.94 (t, *J* = 7.1 Hz, 3H, CH_3_); ^13^C-NMR (DMSO-*d*_6_), δ: 165.07, 154.63, 151.51, 132.74, 131.45, 129.89, 127.84, 127.37, 126.76, 123.45, 122.85, 120.71, 118.69, 118.32, 114.38, 67.27, 30.75, 18.67, 13.62; HR-MS: for C_21_H_21_NO_3_ [M + H]^+^ calculated 336.15942 *m*/*z*, found 336.15990 *m*/*z*.

*2-Hydroxy-N-[2-(prop-2-yloxy)phenyl]naphthalene-1-carboxamide* (**6a**). Yield 72%; Mp 141 °C; IR (cm^−1^): 3397 (ν NH), 1624 (ν C=O), 1533 (δ NH), 1396 (δ COH), 1285 (ν CN), 1115 (ν C_Ar_CO); ^1^H-NMR (DMSO-*d*_6_), δ: 10.57 (s, 1H, OH), 9.42 (s, 1H, NH), 8.25 (d, *J* = 8.1 Hz, 1H, ArH_Phe_), 8.20 (d, *J* = 8.8 Hz, 1H, ArH_Naph_), 7.89 (d, *J* = 8.8 Hz, 1H, ArH_Naph_), 7.85 (d, *J* = 8.1 Hz, 1H, ArH_Naph_), 7.48 (td, *J* = 7.5 Hz, *J* = 1.5 Hz, 1H, ArH_Naph_), 7.36 (td, *J* = 7.0 Hz, *J* = 0.7 Hz, 1H, ArH_Naph_), 7.28 (d, *J* = 8.8 Hz, 1H, ArH_Naph_), 7.11 (d, *J* = 4.0 Hz, 2H, ArH_Phe_), 6.99 (td, *J* = 7.9 Hz, *J* = 3.7 Hz, 1H, ArH_Phe_), 4.68 (sep, *J* = 6.0 Hz, 1H, CH), 1.29 (d, *J* = 6.2 Hz, 6H, CH_3_); ^13^C-NMR (DMSO-*d*_6_), δ: 164.89, 152.57, 142.50, 131.84, 131.24, 128.64, 127.96, 127.79, 126.87, 124.26, 124.24, 123.04, 121.54, 120.36, 118.22, 116.23, 113.79, 70.67, 21.75; HR-MS: for C_20_H_19_NO_3_ [M + H]^+^ calculated 322.14377 *m*/*z*, found 322.14432 *m*/*z*.

*2-Hydroxy-N-[3-(prop-2-yloxy)phenyl]naphthalene-1-carboxamide* (**6b**). Yield 44%; Mp 102 °C; IR (cm^−1^): 3352 (ν NH), 1628 (ν C=O), 1527 (δ NH), 1319 (δ COH), 1276 (ν CN), 1184 (ν C_Ar_CO); ^1^H-NMR (DMSO-*d*_6_), δ: 10.33 (s, 1H, NH), 10.11 (br. s, 1H, OH), 7.85 (d, *J* = 9.2 Hz, 2H, ArH_Naph_), 7.67 (d, *J* = 8.0 Hz, 1H, ArH_Naph_), 7.49 (s, 1H, ArH_Phe_), 7.46 (td, *J* = 6.8 Hz, *J* = 1.7 Hz, 1H, ArH_Naph_), 7.36–7.17 (m, 4H, ArH_Naph_, ArH_Phe_), 6.65 (ddd, *J* = 8.1 Hz, *J* = 2.6 Hz, *J* = 1.6 Hz, 1H, ArH_Phe_), 4.57 (sep, *J* = 6.1 Hz, 1H, CH), 1.28 (d, *J* = 6.2 Hz, 6H, CH_3_); ^13^C-NMR (DMSO-*d*_6_), δ: 165.71, 157.70, 151.61, 140.81, 131.39, 130.08, 129.40, 127.91, 127.37, 126.91, 123.39, 122.94, 118.58, 118.36, 111.45, 110.33, 106.85, 69.13, 21.84; HR-MS: for C_20_H_19_NO_3_ [M + H]^+^ calculated 322.14377 *m*/*z*, found 322.14438 *m*/*z*.

*2-Hydroxy-N-[4-(prop-2-yloxy)phenyl]naphthalene-1-carboxamide* (**6c**). Yield 75%; Mp 133 °C; IR (cm^−1^): 3265 (ν NH), 1625 (ν C=O), 1534 (δ NH), 1435 (δ COH), 1236 (ν CN), 1179 (ν C_Ar_CO); ^1^H-NMR (DMSO-*d*_6_), δ: 10.22 (s, 1H, NH), 10.08 (br. s, 1H, OH), 7.84 (d, *J* = 8.8 Hz, 2H, ArH_Naph_), 7.70 (d, *J* = 8.8 Hz, 2H, ArH_Phe_), 7.69 (d, *J* = 9.2 Hz, 1H, ArH_Naph_), 7.46 (td, *J* = 7.5 Hz, *J* = 1.1 Hz, 1H, ArH_Naph_), 7.32 (td, *J* = 7.3 Hz, *J* = 2.0 Hz, 1H, ArH_Naph_), 7.24 (d, *J* = 9.2 Hz, 1H, ArH_Naph_), 6.91 (d, *J* = 9.2 Hz, 2H, ArH_Phe_), 4.57 (sep, *J* = 6.0 Hz, 1H, CH), 1.26 (d, *J* = 5.9 Hz, 6H, CH_3_); ^13^C-NMR (DMSO-*d*_6_), δ: 165.06, 153.28, 151.49, 132.72, 131.43, 129.86, 127.81, 127.35, 126.75, 123.44, 122.83, 120.72, 118.69, 118.31, 115.84, 69.38, 21.78; HR-MS: for C_20_H_19_NO_3_ [M + H]^+^ calculated 322.14377 *m*/*z*, found 322.14426 *m*/*z*.

*N-[2-(But-2-yloxy)phenyl]-2-hydroxynaphthalene-1-carboxamide* (**7a**). Yield 60%; Mp 48 °C; IR (cm^−1^): 3398 (ν NH), 1624 (ν C=O), 1521 (δ NH), 1396 (δ COH), 1285 (ν CN), 1112 (ν C_Ar_CO); ^1^H-NMR (DMSO-*d*_6_), δ: 10.56 (s, 1H, OH), 9.40 (s, 1H, NH), 8.26 (d, *J* = 8.1 Hz, 1H, ArH_Phe_), 8.19 (d, *J* = 8.4 Hz, 1H, ArH_Naph_), 7.89 (d, *J* = 9.2 Hz, 1H, ArH_Naph_), 7.84 (d, *J* = 8.1 Hz, 1H, ArH_Naph_), 7.48 (td, *J* = 7.9 Hz, *J* = 1.5 Hz, 1H, ArH_Naph_), 7.34 (td, *J* = 8.1 Hz, *J* = 1.1 Hz, 1H, ArH_Naph_), 7.27 (d, *J* = 8.8 Hz, 1H, ArH_Naph_), 7.10 (d, *J* = 4.0 Hz, 2H, ArH_Phe_), 6.98 (td, *J* = 8.2 Hz, *J* = 4.0 Hz, 1H, ArH_Phe_), 4.87 (sx, *J* = 6.0 Hz, 1H, CH), 1.70-1.56 (m, 2H, CH_2_), 1.24 (d, *J* = 6.2 Hz, 3H, CH_3_), 0.91 (t, *J* = 7.3 Hz, 3H, CH_3_); ^13^C-NMR (DMSO-*d*_6_), δ: 164.94, 152.66, 147.72, 131.90, 131.31, 128.55, 128.02, 127.81, 126.91, 124.33, 124.23, 123.10, 121.54, 120.27, 118.28, 116.25, 113.47, 75.35, 28.50, 18.99, 9.47; HR-MS: for C_21_H_21_NO_3_ [M + H]^+^ calculated 336.15942 *m*/*z*, found 336.15982 *m*/*z*.

*N-[3-(But-2-yloxy)phenyl]-2-hydroxynaphthalene-1-carboxamide* (**7b**). Yield 45%; Mp 85 °C; IR (cm^−1^): 3284 (ν NH), 1627 (ν C=O), 1529 (δ NH), 1434 (δ COH), 1275 (ν CN), 1155 (ν C_Ar_CO); ^1^H-NMR (DMSO-*d*_6_), δ: 10.33 (s, 1H, NH), 10.10 (s, 1H, OH), 7.85 (d, *J* = 9.2 Hz, 2H, ArH_Naph_), 7.68 (d, *J* = 8.4 Hz, 1H, ArH_Naph_), 7.50 (t, *J* = 2.2 Hz, 1H, ArH_Phe_), 7.46 (td, *J* = 7.7 Hz, *J* = 1.5 Hz, 1H, ArH_Naph_), 7.36–7.17 (m, 4H, ArH_Naph_, ArH_Phe_), 6.65 (ddd, *J* = 8.1 Hz, *J* = 1.1 Hz, *J* = 0.9 Hz, 1H, ArH_Phe_), 4.33 (sx, *J* = 6.0 Hz, 1H, CH), 1.72-1.48 (m, 2H, CH_2_), 1.25 (d, *J* = 5.9 Hz, 3H, CH_3_), 0.94 (t, *J* = 7.3 Hz, 3H, CH_3_); ^13^C-NMR (DMSO-*d*_6_), δ: 165.74, 158.06, 151.63, 140.84, 131.40, 130.10, 129.43, 127.93, 127.37, 126.93, 123.41, 122.97, 118.60, 118.37, 111.41, 110.36, 106.79, 74.14, 28.58, 19.09, 9.56; HR-MS: for C_21_H_21_NO_3_ [M + H]^+^ calculated 336.15942 *m*/*z*, found 336.15972 *m*/*z*.

*N-[4-(But-2-yloxy)phenyl]-2-hydroxynaphthalene-1-carboxamide* (**7c**). Yield 45%; Mp 139 °C; IR (cm^−1^): 3257 (ν NH), 1621 (ν C=O), 1505 (δ NH), 1401 (δ COH), 1232 (ν CN), 1136 (ν C_Ar_CO); ^1^H-NMR (DMSO-*d*_6_), δ: 10.23 (s, 1H, NH), 10.07 (s, 1H, OH), 7.84 (d, *J* = 8.8 Hz, 2H, ArH_Naph_), 7.70 (d, *J* = 9.2 Hz, 2H, ArH_Phe_), 7.69 (d, *J* = 7.0 Hz, 1H, ArH_Naph_), 7.46 (td, *J* = 7.0 Hz, *J* = 1.1 Hz, 1H, ArH_Naph_), 7.32 (td, *J* = 7.5 Hz, *J* = 2.0 Hz, 1H, ArH_Naph_), 7.24 (d, *J* = 8.8 Hz, 1H, ArH_Naph_), 6.91 (d, *J* = 8.8 Hz, 2H, ArH_Phe_), 4.34 (sx, *J* = 5.9 Hz, 1H, CH), 1.72–1.48 (m, 2H, CH_2_), 1.22 (d, *J* = 5.9 Hz, 3H, CH_3_), 0.93 (t, *J* = 7.3 Hz, 3H, CH_3_); ^13^C-NMR (DMSO-*d*_6_), δ: 165.13, 153.68, 151.57, 132.77, 131.49, 129.96, 127.91, 127.40, 126.84, 123.50, 122.92, 120.78, 118.73, 118.37, 115.90, 74.43, 28.53, 19.05, 9.50; HR-MS: for C_21_H_21_NO_3_ [M + H]^+^ calculated 336.15942 *m*/*z*, found 336.15974 *m*/*z*.

*1-Hydroxy-N-phenylnaphthalene-2-carboxamide* (**8**, CAS 13545-65-8), *1-hydroxy-N-(2-methoxyphenyl)naphtha-lene-2-carboxamide* (**9a**, CAS 26675-52-5), *1-hydroxy-N-(3-methoxyphenyl)naphthalene-2-carboxamide* (**9b**, CAS 110677-79-7) and *1-hydroxy-N-(4-methoxyphenyl)naphthalene-2-carboxamide* (**9c**, CAS 98621-48-8) were synthesized and characterized recently [11].

*N-(2-Ethoxyphenyl)-1-hydroxynaphthalene-2-carboxamide* (**10a**, CAS 26639-37-2). Yield 55%; Mp 139 °C (140–142 °C) [41]; IR (cm^−1^): 3424 (ν NH), 1621 (ν C=O), 1538 (δ NH), 1388 (δ COH), 1288 (ν CN), 1117 (ν C_Ar_CO); ^1^H-NMR (DMSO-*d*_6_), δ: 13.75 (s, 1H, OH), 10.27 (s, 1H, NH), 8.32 (d, *J* = 8.2 Hz, 1H, ArH_Naph_), 8.07 (d, *J* = 8.8 Hz, 1H, ArH_Naph_), 7.91 (d, *J* = 8.1 Hz, 1H, ArH_Naph_), 7.73 (dd, *J* = 7.8 Hz, *J* = 1.2 Hz, 1H, ArH_Phe_), 7.70–7.62 (m, 1H, ArH_Naph_), 7.62–7.54 (m, 1H, ArH_Naph_), 7.47 (d, *J* = 8.8 Hz, 1H, ArH_Naph_), 7.27–7.18 (m, 1H, ArH_Phe_), 7.15–7.08 (m, 1H, ArH_Phe_), 7.00 (t, *J* = 7.6 Hz, 1H, ArH_Phe_), 4.11 (q, *J* = 6.9 Hz, 2H, CH_2_), 1.34 (t, *J* = 6.9 Hz, 3H, CH_3_); ^13^C-NMR (DMSO-*d*_6_), δ: 168.30, 158.61, 151.43, 135.91, 128.82, 127.50, 126.49, 126.08, 125.82, 125.26, 124.85, 123.41, 123.04, 120.27, 118.32, 112.73, 108.78, 63.96, 14.57; HR-MS: for C_19_H_17_NO_3_ [M + H]^+^ calculated 308.12812 *m*/*z*, found 308.12875 *m*/*z*.

*N-(3-Ethoxyphenyl)-1-hydroxynaphthalene-2-carboxamide* (**10b**). Yield 86%; Mp 130 °C; IR (cm^−1^): 3436 (ν NH), 1624 (ν C=O), 1532 (δ NH), 1387 (δ COH), 1293 (ν CN), 1174 (ν C_Ar_CO); ^1^H-NMR (DMSO-*d_6_*), δ: 14.00 (br. s, 1H, OH), 10.41 (s, 1H, NH), 8.31 (d, *J* = 8.3 Hz, 1H, ArH_Naph_), 8.12 (d, *J* = 9.0 Hz, 1H, ArH_Naph_), 7.92 (d, *J* = 7.9 Hz, 1H, ArH_Naph_), 7.67 (ddd, *J* = 8.1 Hz, *J* = 6.9 Hz, *J* = 1.3 Hz, 1H, ArH_Naph_), 7.62–7.54 (m, 1H, ArH_Naph_), 7.47 (d, *J* = 8.8 Hz, 1H, ArH_Naph_), 7.41–7.33 (m, 2H, ArH_Phe_), 7.32–7.26 (m, 1H, ArH_Phe_), 6.76 (ddd, *J* = 7.8 Hz, *J* = 2.4 Hz, *J* = 1.5 Hz, 1H, ArH_Phe_), 4.04 (q, *J* = 7.0 Hz, 2H, CH_2_), 1.35 (t, *J* = 7.0 Hz, 3H, CH_3_); ^13^C-NMR (DMSO-*d*_6_), δ: 169.41, 159.88, 158.62, 138.73, 135.94, 129.36, 129.07, 127.41, 125.85, 124.62, 123.00, 123.00, 117.73, 114.01, 110.77, 108.20, 107.55, 63.02, 14.57; HR-MS: for C_19_H_17_NO_3_ [M + H]^+^ calculated 308.12812 *m*/*z*, found 308.12851 *m*/*z*.

*N-(4-Ethoxyphenyl)-1-hydroxynaphthalene-2-carboxamide* (**10c**, CAS 109883-65-0). Yield 56%; Mp 152 °C (154–155 °C) [41]; IR (cm^−1^): 3436 (ν NH), 1621 (ν C=O), 1536 (δ NH), 1389 (δ COH), 1299 (ν CN), 1146 (ν C_Ar_CO); ^1^H-NMR (DMSO-*d*_6_), δ: 14.21 (br. s, 1H, OH), 10.39 (s, 1H, NH), 8.30 (d, *J* = 8.3 Hz, 1H, ArH_Naph_), 8.11 (d, *J* = 9.0 Hz, 1H, ArH_Naph_), 7.91 (d, *J* = 8.1 Hz, 1H, ArH_Naph_), 7.71–7.52 (m, 4H, ArH_Naph_, ArH_Phe_), 7.45 (d, *J* = 9.0 Hz, 1H, ArH_Naph_), 6.96 (d, *J* = 9.0 Hz, 2H, ArH_Phe_), 4.03 (q, *J* = 6.9 Hz, 2H, CH_2_), 1.33 (t, *J* = 7.0 Hz, 3H, CH_3_); ^13^C-NMR (DMSO-*d*_6_), δ: 170.62, 161.37, 157.15, 137.35, 131.73, 130.43, 128.90, 127.27, 126.15, 125.30, 124.48, 124.40, 119.14, 115.79, 108.90, 64.60, 16.07; HR-MS: for C_19_H_17_NO_3_ [M + H]^+^ calculated 308.12812 *m*/*z*, found 308.12869 *m*/*z*.

*1-Hydroxy-N-(2-propoxyphenyl)naphthalene-2-carboxamide* (**11a**). Yield 55%; Mp 108 °C; IR (cm^−1^): 3440 (ν NH), 1625 (ν C=O), 1537 (δ NH), 1390 (δ COH), 1240 (ν CN), 1110 (ν C_Ar_CO); ^1^H-NMR (DMSO-*d*_6_), δ: 13.83 (br. s, 1H, OH), 10.26 (s, 1H, NH), 8.35–8.28 (m, 1H, ArH_Naph_), 8.06 (d, *J* = 8.8 Hz, 1H, ArH_Naph_), 7.91 (d, *J* = 7.7 Hz, 1H, ArH_Naph_), 7.74–7.55 (m, 3H, ArH_Naph_, ArH_Phe_), 7.47 (d, *J* = 8.8 Hz, 1H, ArH_Naph_), 7.28–7.20 (m, 1H, ArH_Phe_), 7.15–7.09 (m, 1H, ArH_Phe_), 7.00 (td, *J* = 7.6 Hz, *J* = 1.4 Hz, 1H, ArH_Phe_), 4.00 (t, *J* = 6.4 Hz, 2H, CH_2_), 1.74 (sx, *J* = 7.0 Hz, 2H, CH_2_), 0.95 (t, *J* = 7.4 Hz, 3H, CH_3_); ^13^C-NMR (DMSO-*d*_6_), δ: 168.49, 158.78, 151.86, 135.90, 128.84, 128.49, 126.68, 125.97, 125.80, 125.56, 124.80, 123.26, 123.03, 120.22, 118.25, 112.70, 108.53, 69.66, 22.01, 10.25; HR-MS: for C_20_H_19_NO_3_ [M + H]^+^ calculated 322.14377 *m*/*z*, found 322.14432 *m*/*z*.

*1-Hydroxy-N-(3-propoxyphenyl)naphthalene-2-carboxamide* (**11b**). Yield 58%; Mp 95 °C; IR (cm^−1^): 3436 (ν NH), 1627 (ν C=O), 1533 (δ NH), 1330 (δ COH), 1292 (ν CN), 1173 (ν C_Ar_CO); ^1^H-NMR (DMSO-*d*_6_), δ: 14.00 (s, 1H, OH), 10.40 (s, 1H, NH), 8.31 (d, *J* = 8.2 Hz, 1H, ArH_Naph_), 8.12 (d, *J* = 9.0 Hz, 1H, ArH_Naph_), 7.92 (d, *J* = 8.1 Hz, 1H, ArH_Naph_), 7.67 (ddd, *J* = 8.1 Hz, *J* = 6.9 Hz, *J* = 1.3 Hz, 1H, ArH_Naph_), 7.62–7.54 (m, 1H, ArH_Naph_), 7.47 (d, *J* = 8.8 Hz, 1H, ArH_Naph_), 7.42–7.33 (m, 2H, ArH_Phe_), 7.33–7.25 (m, 1H, ArH_Phe_), 6.80–6.73 (m, 1H, ArH_Phe_), 3.94 (t, *J* = 6.5 Hz, 2H, CH_2_), 1.75 (sx, *J* = 7.0 Hz, 2H, CH_2_), 0.99 (t, *J* = 7.3 Hz, 3H, CH_3_); ^13^C-NMR (DMSO-*d*_6_), δ: 169.41, 159.88, 158.81, 138.73, 135.94, 129.36, 129.05, 127.41, 125.85, 124.62, 123.00, 123.00, 117.73, 114.00, 110.78, 108.23, 107.55, 68.97, 21.96, 10.31; HR-MS: for C_20_H_19_NO_3_ [M + H]^+^ calculated 322.14377 *m*/*z*, found 322.14426 *m*/*z*.

*1-Hydroxy-N-(4-propoxyphenyl)naphthalene-2-carboxamide* (**11c**). Yield 65%; Mp 127 °C; IR (cm^−1^): 3415 (ν NH), 1622 (ν C=O), 1536 (δ NH), 1389 (δ COH), 1297 (ν CN), 1151 (ν C_Ar_CO); ^1^H-NMR (DMSO-*d*_6_), δ: 14.21 (br. s, 1H, OH), 10.39 (s, 1H, NH), 8.30 (d, *J* = 8.4 Hz, 1H, ArH_Naph_), 8.11 (d, *J* = 9.0 Hz, 1H, ArH_Naph_), 7.91 (d, *J* = 7.9 Hz, 1H, ArH_Naph_), 7.70–7.54 (m, 4H, ArH_Naph_, ArH_Phe_), 7.45 (d, *J* = 8.8 Hz, 1H, ArH_Naph_), 7.01–6.93 (m, 2H, ArH_Phe_), 3.93 (t, *J* = 6.5 Hz, 2H, CH_2_), 1.73 (sx, *J* = 7.1 Hz, 2H, CH_2_), 0.98 (t, *J* = 7.4 Hz, 3H, CH_3_); ^13^C-NMR (DMSO-*d*_6_), δ: 169.09, 159.85, 155.80, 135.83, 130.22, 129.92, 127.37, 125.76, 124.64, 123.77, 122.95, 122.88, 117.61, 114.32, 107.38, 69.06, 21.96, 10.28; HR-MS: for C_20_H_19_NO_3_ [M + H]^+^ calculated 322.14377 *m*/*z*, found 322.14419 *m*/*z*.

*N-(2-Butoxyphenyl)-1-hydroxynaphthalene-2-carboxamide* (**12a**). Yield 61%; Mp 112 °C; IR (cm^−1^): 3443 (ν NH), 1626 (ν C=O), 1538 (δ NH), 1390 (δ COH), 1253 (ν CN), 1111 (ν C_Ar_CO); ^1^H-NMR (DMSO-*d*_6_), δ: 13.82 (s, 1H, OH), 10.23 (s, 1H, NH), 8.31 (d, *J* = 8.2 Hz, 1H, ArH_Naph_), 8.05 (d, *J* = 9.0 Hz, 1H, ArH_Naph_), 7.92 (d, *J* = 7.9 Hz, 1H, ArH_Naph_), 7.73–7.63 (m, 2H, ArH_Naph_, ArH_Phe_), 7.62–7.55 (m, 1H, ArH_Naph_), 7.47 (d, *J* = 8.8 Hz, 1H, ArH_Naph_), 7.28–7.20 (m, 1H, ArH_Phe_), 7.15–7.09 (m, 1H, ArH_Phe_), 7.00 (td, *J* = 7.6 Hz, *J* = 1.1 Hz, 1H, ArH_Phe_), 4.04 (t, *J* = 6.3 Hz, 2H, CH_2_), 1.70 (qi, *J* = 7.3 Hz, 2H, CH_2_), 1.41 (sx, *J* = 7.4 Hz, 2H, CH_2_), 0.86 (t, *J* = 7.3 Hz, 3H, CH_3_); ^13^C-NMR (DMSO-*d*_6_), δ: 168.50, 158.79, 151.86, 135.88, 128.81, 127.47, 126.67, 125.99, 125.79, 125.53, 124.82, 123.23, 123.01, 120.21, 118.20, 112.67, 108.52, 67.87, 30.64, 18.53, 13.52; HR-MS: for C_21_H_21_NO_3_ [M + H]^+^ calculated 336.15942 *m*/*z*, found 336.15994 *m*/*z*.

*N-(3-Butoxyphenyl)-1-hydroxynaphthalene-2-carboxamide* (**12b**). Yield 58%; Mp 107 °C; IR (cm^−1^): 3288 (ν NH), 1633 (ν C=O), 1527 (δ NH), 1389 (δ COH), 1291 (ν CN), 1170 (ν C_Ar_CO); ^1^H-NMR (DMSO-*d*_6_), δ: 14.00 (s, 1H, OH), 10.40 (s, 1H, NH), 8.31 (dt, *J* = 8.2 Hz, *J* = 0.6 Hz, 1H, ArH_Naph_), 8.12 (d, *J* = 8.8 Hz, 1H, ArH_Naph_), 7.92 (d, *J* = 7.9 Hz, 1H, ArH_Naph_), 7.71–7.63 (m, 1H, ArH_Naph_), 7.62–7.54 (m, 1H, ArH_Naph_), 7.47 (d, *J* = 8.8 Hz, 1H, ArH_Naph_), 7.42–7.33 (m, 2H, ArH_Phe_), 7.32–7.25 (m, 1H, ArH_Phe_), 6.76 (ddd, *J* = 7.9 Hz, *J* = 2.5 Hz, *J* = 1.3 Hz, 1H, ArH_Phe_), 3.98 (t, *J* = 6.5 Hz, 2H, CH_2_), 1.72 (qi, *J* = 7.3 Hz, 2H, CH_2_), 1.45 (sx, *J* = 7.4 Hz, 2H, CH_2_), 0.94 (t, *J* = 7.5 Hz, 3H, CH_3_); ^13^C-NMR (DMSO-*d*_6_), δ: 169.34, 159.88, 158.81, 138.72, 135.94, 129.34, 129.05, 127.41, 125.84, 124.62, 123.00, 123.00, 117.73, 114.00, 110.77, 108.23, 107.54, 67.15, 30.67, 18.67, 13.60; HR-MS: for C_21_H_21_NO_3_ [M+H]^+^ calculated 336.15942 *m*/*z*, found 336.15990 *m*/*z*.

*N-(4-Butoxyphenyl)-1-hydroxynaphthalene-2-carboxamide* (**12c**). Yield 57%; Mp 124 °C; IR (cm^−1^): 3328 (ν NH), 1633 (ν C=O), 1532 (δ NH), 1391 (δ COH), 1296 (ν CN), 1149 (ν C_Ar_CO); ^1^H-NMR (DMSO-*d*_6_), δ: 14.22 (br. s, 1H, OH), 10.39 (s, 1H, NH), 8.30 (d, *J* = 8.2 Hz, 1H, ArH_Naph_), 8.11 (d, *J* = 9.0 Hz, 1H, ArH_Naph_), 7.91 (d, *J* = 7.9 Hz, 1H, ArH_Naph_), 7.71–7.53 (m, 4H, ArH_Naph_, ArH_Phe_), 7.45 (d, *J* = 8.8 Hz, 1H, ArH_Naph_), 6.97 (d, *J* = 9.0 Hz, 2H, ArH_Phe_), 3.97 (t, *J* = 6.4 Hz, 2H, CH_2_), 1.70 (qi, *J* = 7.3 Hz, 2H, CH_2_), 1.44 (sx, *J* = 7.4 Hz, 2H, CH_2_), 0.94 (t, *J* = 7.4 Hz, 3H, CH_3_); ^13^C-NMR (DMSO-*d*_6_), δ: 169.11, 159.87, 155.82, 135.85, 130.22, 128.93, 127.38, 125.77, 124.65, 123.77, 122.97, 122.98, 117.63, 114.32, 107.40, 67.27, 30.70, 18.65, 13.58; HR-MS: for C_21_H_21_NO_3_ [M + H]^+^ calculated 336.15942 *m*/*z*, found 336.15973 *m*/*z*.

*1-Hydroxy-N-[2-(prop-2-yloxy)phenyl]naphthalene-2-carboxamide* (**13a**). Yield 49%; Mp 123 °C; IR (cm^−1^): 3425 (ν NH), 1629 (ν C=O), 1538 (δ NH), 1391 (δ COH), 1289 (ν CN), 1115 (ν C_Ar_CO); ^1^H-NMR (DMSO-*d*_6_), δ: 13.59 (s, 1H, OH), 10.27 (s, 1H, NH), 8.33 (d, *J* = 8.4 Hz, 1H, ArH_Naph_), 8.06 (d, *J* = 9.0 Hz, 1H, ArH_Naph_), 7.92 (d, *J* = 7.9 Hz, 1H, ArH_Naph_), 7.81 (dd, *J* = 7.9 Hz, *J* = 1.5 Hz, 1H, ArH_Phe_), 7.67 (td, *J* = 7.4 Hz, *J* = 1.5 Hz, 1H, ArH_Naph_), 7.63–7.55 (m, 1H, ArH_Naph_), 7.49 (d, *J* = 8.8 Hz, 1H, ArH_Naph_), 7.24–7.11 (m, 1H, ArH_Phe_), 7.16–7.11 (m, 1H, ArH_Phe_), 6.99 (td, *J* = 7.6 Hz, *J* = 1.6 Hz, 1H, ArH_Phe_), 4.63 (sep, *J* = 6.0 Hz, 1H, CH), 1.30 (d, *J* = 6.0 Hz, 6H, CH_3_); ^13^C-NMR (DMSO-*d*_6_), δ: 167.94, 158.23, 150.13, 135.90, 128.76, 127.50, 127.20, 126.18, 125.79, 124.94, 124.86, 123.48, 123.04, 120.37, 118.46, 114.46, 109.16, 70.94, 21.82; HR-MS: for C_20_H_19_NO_3_ [M + H]^+^ calculated 322.14377 *m*/*z*, found 322.14430 *m*/*z*.

*1-Hydroxy-N-[3-(prop-2-yloxy)phenyl]naphthalene-2-carboxamide* (**13b**). Yield 77%; Mp 154 °C; IR (cm^−1^): 3301 (ν NH), 1633 (ν C=O), 1530 (δ NH), 1379 (δ COH), 1298 (ν CN), 1156 (ν C_Ar_CO); ^1^H-NMR (DMSO-*d*_6_), δ: 14.00 (s, 1H, OH), 10.39 (s, 1H, NH), 8.31 (d, *J* = 7.9 Hz, 1H, ArH_Naph_), 8.12 (d, *J* = 9.0 Hz, 1H, ArH_Naph_), 7.92 (d, *J* = 7.9 Hz, 1H, ArH_Naph_), 7.67 (ddd, *J* = 8.1 Hz, *J* = 6.9 Hz, *J* = 1.5 Hz, 1H, ArH_Naph_), 7.62–7.55 (m, 1H, ArH_Naph_), 7.47 (d, *J* = 9.0 Hz, 1H, ArH_Naph_), 7.39–7.25 (m, 3H, ArH_Phe_), 6.77–6.72 (m, 1H, ArH_Phe_), 4.60 (sep, *J* = 6.0 Hz, 1H, CH), 1.30 (d, *J* = 6.0 Hz, 6H, CH_3_); ^13^C-NMR (DMSO-*d*_6_), δ: 169.40, 159.87, 157.58, 138.75, 135.93, 129.36, 129.05, 127.41, 125.85, 124.62, 123.00, 123.00, 117.71, 113.94, 111.97, 109.39, 107.51, 69.30, 21.78; HR-MS: for C_20_H_19_NO_3_ [M + H]^+^ calculated 322.14377 *m*/*z*, found 322.14438 *m*/*z*.

*1-Hydroxy-N-[4-(prop-2-yloxy)phenyl]naphthalene-2-carboxamide* (**13c**). Yield 66%; Mp 173 °C; IR (cm^−1^): 3360 (ν NH), 1623 (ν C=O), 1528 (δ NH), 1379 (δ COH), 1300 (ν CN), 1157 (ν C_Ar_CO); ^1^H-NMR (DMSO-*d*_6_), δ: 14.22 (s, 1H, OH), 10.38 (s, 1H, NH), 8.30 (d, *J* = 8.1 Hz, 1H, ArH_Naph_), 8.11 (d, *J* = 8.8 Hz, 1H, ArH_Naph_), 7.91 (d, *J* = 8.1 Hz, 1H, ArH_Naph_), 7.71–7.53 (m, 4H, ArH_Naph_, ArH_Phe_), 7.45 (d, *J* = 8.8 Hz, 1H, ArH_Naph_), 6.95 (d, *J* = 9.0 Hz, 2H, ArH_Phe_), 4.59 (sep, *J* = 6.0 Hz, 1H, CH), 1.27 (d, *J* = 6.0 Hz, 6H, CH_3_); ^13^C-NMR (DMSO-*d*_6_), δ: 169.11, 159.85, 154.56, 135.85, 130.16, 128.93, 127.38, 125.79, 124.65, 123.83, 122.97, 122.89, 117.64, 115.61, 107.40, 69.38, 21.76; HR-MS: for C_20_H_19_NO_3_ [M + H]^+^ calculated 322.14377 *m*/*z*, found 322.14422 *m*/*z*.

*N-[2-(But-2-yloxy)phenyl]-1-hydroxynaphthalene-2-carboxamide* (**14a**). Yield 56%; Mp 60 °C; IR (cm^−1^): 3418 (ν NH), 1627 (ν C=O), 1540 (δ NH), 1389 (δ COH), 1289 (ν CN), 1112 (ν C_Ar_CO); ^1^H-NMR (DMSO-*d*_6_), δ: 13.66 (s, 1H, OH), 10.26 (s, 1H, NH), 8.35–8.29 (m, 1H, ArH_Naph_), 8.05 (d, *J* = 9.0 Hz, 1H, ArH_Naph_), 7.92 (d, *J* = 7.5 Hz, 1H, ArH_Naph_), 7.77 (dd, *J* = 7.9 Hz, *J* = 1.5 Hz, 1H, ArH_Phe_), 7.70–7.63 (m, 1H, ArH_Naph_), 7.62–7.55 (m, 1H, ArH_Naph_), 7.48 (d, *J* = 8.6 Hz, 1H, ArH_Naph_), 7.25–7.18 (m, 1H, ArH_Phe_), 7.15–7.09 (m, 1H, ArH_Phe_), 6.98 (td, *J* = 7.6 Hz, *J* = 1.5 Hz, 1H, ArH_Phe_), 4.43 (sx, *J* = 6.0 Hz, 1H, CH), 1.76–1.51 (m, 2H, CH_2_), 1.25 (d, *J* = 6.0 Hz, 3H, CH_3_), 0.90 (t, *J* = 7.4 Hz, 3H, CH_3_); ^13^C-NMR (DMSO-*d*_6_), δ: 168.09, 158.40, 150.52, 135.88, 128.76, 127.49, 127.08, 126.32, 125.77, 125.21, 124.85, 123.39, 123.04, 120.25, 118.37, 114.29, 108.98, 75.54, 28.53, 18.85, 9.22; HR-MS: for C_21_H_21_NO_3_ [M + H]^+^ calculated 336.15942 *m*/*z*, found 336.15982 *m*/*z*.

*N-[3-(But-2-yloxy)phenyl]-1-hydroxynaphthalene-2-carboxamide* (**14b**). Yield 59%; Mp 97 °C; IR (cm^−1^): 3324 (ν NH), 1602 (ν C=O), 1526 (δ NH), 1329 (δ COH), 1296 (ν CN), 1153 (ν C_Ar_CO); ^1^H-NMR (DMSO-*d*_6_), δ: 14.00 (s, 1H, OH), 10.38 (s, 1H, NH), 8.35–8.28 (m, 1H, ArH_Naph_), 8.12 (d, *J* = 9.0 Hz, 1H, ArH_Naph_), 7.92 (d, *J* = 7.9 Hz, 1H, ArH_Naph_), 7.67 (ddd, *J* = 8.1 Hz, *J* = 6.8 Hz, *J* = 1.4 Hz, 1H, ArH_Naph_), 7.62–7.54 (m, 1H, ArH_Naph_), 7.47 (d, *J* = 9.0 Hz, 1H, ArH_Naph_), 7.40–7.31 (m, 2H, ArH_Phe_), 7.31–7.24 (m 1H, ArH_Phe_), 6.75 (ddd, *J* = 7.8 Hz, *J* = 2.4 Hz, *J* = 1.3 Hz, 1H, ArH_Phe_), 4.38 (sx, *J* = 6.0 Hz, 1H, CH), 1.77-1.52 (m, 2H, CH_2_), 1.26 (d, *J* = 6.0 Hz, 3H, CH_3_), 0.94 (t, *J* = 7.4 Hz, 3H, CH_3_); ^13^C-NMR (DMSO-*d*_6_), δ: 169.40, 159.88, 157.93, 138.78, 135.93, 129.37, 129.05, 127.41, 125.84, 124.62, 123.00, 123.00, 117.72, 113.94, 111.97, 109.40, 107.55, 74.28, 28.52, 18.99, 9.44; HR-MS: for C_21_H_21_NO_3_ [M + H]^+^ calculated 336.15942 *m*/*z*, found 336.15980 *m*/*z*.

*N-[4-(But-2-yloxy)phenyl]-1-hydroxynaphthalene-2-carboxamide* (**14c**). Yield 59%; Mp 149 °C; IR (cm^−1^): 3359 (ν NH), 1623 (ν C=O), 1525 (δ NH), 1386 (δ COH), 1302 (ν CN), 1158 (ν C_Ar_CO); ^1^H-NMR (DMSO-*d*_6_), δ: 14.22 (s, 1H, OH), 10.38 (s, 1H, NH), 8.34–8.26 (m, 1H, ArH_Naph_), 8.11 (d, *J* = 9.0 Hz, 1H, ArH_Naph_), 7.91 (d, *J* = 7.9 Hz, 1H, ArH_Naph_), 7.66 (ddd, *J* = 8.1 Hz, *J* = 6.9 Hz, *J* = 1.5 Hz, 1H, ArH_Naph_), 7.63–7.53 (m, 3H, ArH_Naph_, ArH_Phe_), 7.45 (d, *J* = 8.8 Hz, 1H, ArH_Naph_), 6.99–6.91 (m, 2H, ArH_Phe_), 4.37 (sx, *J* = 6.0 Hz, 1H, CH), 1.75–1.49 (m, 2H, CH_2_), 1.23 (d, *J* = 6.0 Hz, 3H, CH_3_), 0.93 (t, *J* = 7.4 Hz, 3H, CH_3_); ^13^C-NMR (DMSO-*d*_6_), δ: 169.11, 159.87, 154.91, 135.85, 130.14, 128.93, 127.38, 125.77, 124.65, 123.85, 122.97, 122.89, 117.63, 115.64, 107.40, 74.37, 28.48, 18.97, 9.41; HR-MS: for C_21_H_21_NO_3_ [M + H]^+^ calculated 336.15942 *m*/*z*, found 336.15990 *m*/*z*.

### 3.3. In Vitro Antimycobacterial Evaluation

*Mycobacterium tuberculosis* H37Ra ATCC 25177 was grown in Middlebrook broth (MB), supplemented with Oleic-Albumin-Dextrose-Catalase (OADC) supplement (Becton, Dickinson & Comp., Franklin Lakes, NJ, USA) and mycobactin J (2 μg/mL). At log phase growth, a culture sample (10 mL) was centrifuged at 15,000 rpm/20 min using a bench top centrifuge (Model CR 4-12, Jouan Inc., Winchester, VA, USA). Following the removal of the supernatant, the pellet was washed in fresh Middlebrook 7H9GC broth and re-suspended in fresh, ODAC-supplemented MB (10 mL). The turbidity was adjusted to match McFarland standard No. 1 (3 × 10^8^ cfu) with MB broth. A further 1:20 dilution of the culture was then performed in MB broth. The antimicrobial susceptibility of *M. tuberculosis* was investigated in a 96-well plate format. In these experiments, sterile deionised water (300 µL) was added to all outer-perimeter wells of the plates to minimize evaporation of the medium in the test wells during incubation. Each evaluated compound (100 µL) was incubated with *M. tuberculosis* (100 µL). Dilutions of each compound were prepared in duplicate. For all synthesized compounds, final concentrations ranged from 1000 μg/mL to 8 μg/mL. All compounds were dissolved in DMSO, and subsequent dilutions were made in supplemented MB. The plates were sealed with parafilm and incubated at 37 °C for 7 days. Following incubation, a 10% addition of alamarBlue (AbD Serotec, Kidlington, UK) was mixed into each well, and readings at 570 nm and 600 nm were taken, initially for background subtraction and subsequently after 24 h re-incubation. The background subtraction is necessary for strongly coloured compounds, where the colour may interfere with the interpretation of any colour change. For non-interfering compounds, a blue colour in the well was interpreted as the absence of growth, and a pink colour was scored as growth.

The evaluation of the in vitro antimycobacterial activity of the compounds was additionally performed against *Mycobacterium kansasii* DSM 44162 and *M. smegmatis* ATCC 700084. The broth dilution micro-method in Middlebrook 7H9 medium (Difco, Lawrence, KS, USA) supplemented with ADC Enrichment (Becton, Dickinson & Comp.) was used to determine the minimum inhibitory concentration (MIC) as previously described [42]. The compounds were dissolved in DMSO (Sigma-Aldrich), and the final concentration of DMSO did not exceed 2.5% of the total solution composition. The final concentrations of the evaluated compounds ranging from 256 μg/mL to 0.125 μg/mL were obtained by twofold serial dilution of the stock solution in microtiter plate with sterile medium. Bacterial inocula were prepared by transferring colonies from culture to sterile water. The cell density was adjusted to 0.5 McFarland units using a densitometer (Densi-La-Meter, LIAP, Riga, Latvia). The final inoculum was made by 1:1000 dilution of the suspension with sterile water. Drug-free controls, sterility controls and controls consisted of medium and DMSO alone were included. The determination of results was performed visually after 3 days of static incubation in the darkness at 37 °C in an aerobic atmosphere for *M. smegmatis* and after 7 days of static incubation in the darkness at 37 °C in an aerobic atmosphere for *M. kansasii*.

The minimum inhibitory concentration (MIC) was defined as the lowest concentration of the compound at which no visible bacterial growth was observed, i.e., the MIC is the lowest concentration that prevented a visual colour change from blue to pink. MIC is defined for mycobacteria as 90% or greater (IC_90_) reduction of growth in comparison with the control. The MIC/IC_90_ value is routinely and widely used in bacterial assays and is a standard detection limit according to the Clinical and Laboratory Standards Institute (CLSI, www.clsi.org). Rifampicin and isoniazid (Sigma-Aldrich) were used as the standards as they are clinically used antimycobacterial drugs. The results are summarized in Table 1.

### 3.4. MTT Assay

For the MTT (3-(4,5-dimethylthiazol-2-yl)-2,5-diphenyltetrazolium bromide) assay, the outer wells of a 96-well plate were filled with 200 µL of sterile water, and the inner wells were filled with 100 µL of the tested compound at the MIC to be examined. Compounds were prepared as previously stated and diluted in Middlebrook media to achieve the desired concentration. *Mycobacterium tuberculosis* H37Ra ATCC 25177 was suspended in ODAC supplemented Middlebrook broth at a MacFarland standard of 1.0 and then diluted 1:20, using Middlebrook broth as a diluent. The diluted mycobacteria (100 µL) were added to each well containing the compound to be tested. A negative growth control was composed of 100 µL of DMSO and 100 µL of the media, and the diluted mycobacteria in broth absent of inhibiting compounds were used as a positive growth control. All compounds and controls were prepared in triplicate. Plates were incubated at 37 °C for 7 days. After the incubation period, 10% well volume of MTT reagent was mixed into each well and incubated at 37 °C for 24 h. The reagent and media were then aspirated from the wells, to which 50 µL 99% isopropanol was then added, and the plates were read at 570 nm. The absorbance readings from the cells, grown in the presence of the tested compounds, were compared with uninhibited cell growth (using DMSO as the blank) to determine the relative percent viability. The percent viability was determined through the MTT assay. The percent viability is calculated through comparison of a measured value against that of the uninhibited control: %viability = OD_570_E/OD_570_P × 100, where OD_570_E is the reading from the compound-exposed cells, while OD_570_P is the reading from the uninhibited cells (positive control). Cytotoxic potential is determined by a percent viability of <70%.

### 3.5. In Vitro Antiproliferative Assay

Human monocytic leukemia THP-1 cells were used for in vitro antiproliferative assay. Cells were obtained from the European Collection of Cell Cultures (ECACC, Salisbury, UK) and routinely cultured in RPMI 1640 medium supplemented with 10% fetal bovine serum, 2% l-glutamine, 1% penicillin and streptomycin at 37 °C with 5% CO_2_. Cells were passaged at approximately one-week intervals. Antiproliferative activity of the compounds was determined using a Water Soluble Tetrazolium Salts-1 (WST-1, 2-(4-iodophenyl)-3-(4-nitrophenyl)-5-(2,4-disulfophenyl)-2*H*-tetrazolium) assay kit (Roche Diagnostics, Mannheim, Germany) according to the manufacturer’s instructions. The tested compounds were dissolved in DMSO and added in five increasing concentrations (0.37, 1.1, 3.3, 10 and 30 μM) to the cell suspension in the culture RPMI 1640 medium. The maximum concentration of DMSO in the assays never exceeded 0.1%. Subsequently, the cells were incubated for 24 h at 37 °C with 5% CO_2_. For WST-1 assays, cells were seeded into 96-well plates (5 × 10^4^ cells/well in 100 μL culture medium) in triplicate in serum-free RPMI 1640 medium, and measurements were taken 24 h after the treatment with the compounds. The median inhibition concentration values, IC_50_, were deduced through the production of a dose-response curve. All data were evaluated using GraphPad Prism 5.00 software (GraphPad Software, San Diego, CA, USA). The results are summarized in Table 1.

## 4. Conclusions

Series of nineteen *N*-(alkoxyphenyl)-2-hydroxynaphthalene-1-carboxamides (**1**–**7c**) and their nineteen positional isomers *N*-(alkoxyphenyl)-1-hydroxynaphthalene-2-carboxamides (**8**–**14c**) were prepared by means of microwave synthesis and subsequently characterized. All the compounds were tested for their in vitro antimycobacterial activity against *M. tuberculosis*, *M. smegmatis* and *M. kansasii*. The most effective compounds were also tested for their in vitro antiproliferative effect against the THP-1 cells. Lipophilicity was found as the main physicochemical parameter influencing the activity, and more lipophilic compounds expressed higher potency. In general, *N*-(alkoxyphenyl)-2-hydroxynaphthalene-1-carboxamides showed higher activity against *M. tuberculosis*, while *N*-(alkoxyphenyl)-1-hydroxynaphthalene-2-carboxamides demonstrated higher effect against *M. smegmatis* and *M. kansasii*, but also stronger antiproliferative effect against the human THP-1 cell line. 2-Hydroxy-*N*-(4-propoxyphenyl)naphthalene-1-carboxamide (**4c**) showed the highest activity (MIC = 12 µM) against *M. tuberculosis* with insignificant toxicity, while *N*-[3-(but-2-yloxy)phenyl]- (**7b**) and *N*-[4-(but-2-yloxy)phenyl]-2-hydroxynaphthalene-1-carboxamide (**7c**) demonstrated high activity against all three mycobacterial strains and insignificant toxicity. Their potency is comparable with that of rifampicin. The performed MTT assay of the selected most efficient compounds showed that they cause a decrease of mycobacterial cell metabolism. Based on the presented results it can be concluded that some of the discussed amides can be considered as promising agents for subsequent design of novel antimycobacterial agents.

## Figures and Tables

**Table 1:** Structures of discussed ring-substituted hydroxynaphthalenecarboxamides **1**–**14c**; calculated values of log *P*, molar volume (MV (cm^3^)) of R substituents and electronic σ parameters of R substituents; in vitro antimycobacterial activity (MIC) of compounds in comparison with isoniazid (INH) and rifampicin (RIF) standards; in vitro antiproliferative assay (IC_50_) of chosen compounds.

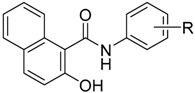								
Compound	R	log *P* ^a^	MV ^a^	σ ^a^	MIC (µM)			IC_50_ (µM)
					MT	MS	MK	
**1**	H	4.49	0	0	94	486	15	>30
**2a**	2-OCH_3_	4.54	37.15	−0.28	852	873	873	-
**2b**	3-OCH_3_	4.51	37.15	0.11	852	218	109	-
**2c**	4-OCH_3_	4.30	37.15	−0.27	852	436	218	-
**3a**	2-OC_2_H_5_	4.88	53.66	−0.29	26	832	832	>30
**3b**	3-OC_2_H_5_	4.83	53.66	0.10	813	416	208	-
**3c**	4-OC_2_H_5_	4.76	53.66	−0.29	26	832	104	>30
**4a**	2-OC_3_H_7_	5.22	70.16	−0.29	37	796	99	>30
**4b**	3-OC_3_H_7_	5.14	70.16	0.14	46	398	199	8.97 ± 0.34
**4c**	4-OC_3_H_7_	5.21	70.16	−0.28	12	398	99	>30
**5a**	2-OC_4_H_9_	5.53	86.67	−0.28	745	763	763	>30
**5b**	3-OC_4_H_9_	5.49	86.67	0.14	23	381	190	>30
**5c**	4-OC_4_H_9_	5.54	86.67	−0.28	745	763	95	>30
**6a**	2-OCH(CH_3_)_2_	5.15	70.54	−0.47	31	796	99	>30
**6b**	3-OCH(CH_3_)_2_	5.06	70.54	0.10	778	398	199	8.84 ± 0.22
**6c**	4-OCH(CH_3_)_2_	5.04	70.54	−0.45	25	796	99	>30
**7a**	2-OCH(CH_3_)CH_2_CH_3_	5.47	87.05	−0.27	44	763	95	8.35 ± 1.25
**7b**	3-OCH(CH_3_)CH_2_CH_3_	5.40	87.05	0.14	23	12	47	>30
**7c**	4-OCH(CH_3_)CH_2_CH_3_	5.40	87.05	−0.27	23	12	95	>30
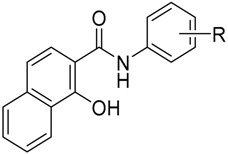								
**Compound**	**R**	**log *P*^a^**	**MV ^a^**	**σ ^a^**	**MIC (µM)**			**IC_50_ (µM)**
					**MT**	**MS**	**MK**	
**8**	H	4.52	0	0	97	243	15	>30
**9a**	2-OCH_3_	4.61	37.15	−0.28	852	873	873	-
**9b**	3-OCH_3_	4.56	37.15	0.11	852	873	873	-
**9c**	4-OCH_3_	4.37	37.15	−0.27	852	873	873	-
**10a**	2-OC_2_H_5_	4.92	53.66	−0.29	813	832	832	-
**10b**	3-OC_2_H_5_	4.88	53.66	0.10	813	832	52	3.02 ± 0.96
**10c**	4-OC_2_H_5_	4.67	53.66	−0.29	813	832	832	-
**11a**	2-OC_3_H_7_	5.26	70.16	−0.29	778	796	24	-
**11b**	3-OC_3_H_7_	5.21	70.16	0.14	24	199	49	1.37 ± 0.72
**11c**	4-OC_3_H_7_	5.27	70.16	−0.28	778	796	24	>30
**12a**	2-OC_4_H_9_	5.60	86.67	−0.28	745	763	763	-
**12b**	3-OC_4_H_9_	5.54	86.67	0.14	23	190	47	1.80 ± 0.32
**12c**	4-OC_4_H_9_	5.60	86.67	−0.28	745	763	23	>30
**13a**	2-OCH(CH_3_)_2_	5.18	70.54	−0.47	778	796	796	-
**13b**	3-OCH(CH_3_)_2_	5.13	70.54	0.10	389	796	796	4.32 ± 0.74
**13c**	4-OCH(CH_3_)_2_	5.11	70.54	−0.45	778	796	49	9.39 ± 1.39
**14a**	2-OCH(CH_3_)CH_2_CH_3_	5.52	87.05	−0.27	745	763	47	9.03 ± 1.18
**14b**	3-OCH(CH_3_)CH_2_CH_3_	5.47	87.05	0.14	23	12	47	1.17 ± 0.38
**14c**	4-OCH(CH_3_)CH_2_CH_3_	5.46	87.05	−0.27	745	12	23	3.41 ± 0.41
**INH**	-	−0.63	-	-	58	117	29	>30
**RIF**	-	2.24	-	-	10	19	0.15	>30

^a^ calculated using ACD/Percepta ver. 2012 (Advanced Chemistry Development, Inc., Toronto, ON, Canada, 2012); MT = *M. tuberculosis* H37Ra ATCC 25177, MS = *Mycobacterium smegmatis* ATCC 700084 and MK = *M. kansasii* DSM 44162.
